# The psychology of volition

**DOI:** 10.1007/s00221-013-3407-6

**Published:** 2013-01-25

**Authors:** Chris Frith

**Affiliations:** 1 Interacting Minds Centre, Aarhus University, Aarhus, Denmark; 2 All Souls College, Oxford, UK; 3 Wellcome Trust Centre for Neuroimaging, UCL, London, UK

**Keywords:** Volition, Agency, Responsibility, Discussion, Culture

## Abstract

Volition can be studied from two perspectives. From the third-person view, volitional behaviour is internally generated, rather than being determined by the immediate environmental context, and is therefore, to some extent, unpredictable. Such behaviour is not unique to humans, since it is seen in many other species including invertebrates. From the first-person view, our experience of volitional behaviour includes a vivid sense of agency. We feel that, through our intentions, we can cause things to happen and we can choose between different actions. Our experience of agency is not direct. It depends on sub-personal inferences derived from prior expectations and sensations associated with movement. As a result, our experiences and intuitions about volition can be unreliable and uncertain. Nevertheless, our experience of agency is not a mere epiphenomenon. Anticipation of the regret we might feel after making the wrong choice can alter behaviour. Furthermore, the strong sense of responsibility, associated with agency, has a critical role in creating social cohesion and group benefits. We can only study the experience of agency in humans who can describe their experiences. The discussion of the experience of volition, that introspection and communication make possible, can change our experience of volitional actions. As a result, agency, regret and responsibility are cultural phenomena that are unique to humans.

## What is a voluntary act?

When we consider the movements that people make, there is a fundamental distinction between reflexes and voluntary acts. Reflex movements occur completely outwith our control. For example, we cannot make our pupil contract by thought alone and we cannot stop it contracting when it is illuminated. In contrast, a voluntary act involves a movement that we can choose to make (or not), deliberately and by thought alone. There are, of course, movements that lie between these two extremes. For example, we can choose to blink, but we cannot stop a blink occurring as a reflexive response to a puff of air to the eye. Nevertheless, we experience most of the actions in our daily repertoire as voluntary.

However, defining voluntary acts as behaviour over which we have control leads to many deep philosophical problems. In what sense do we control our behaviour? Does such control imply the possibility of mental causation and the existence of free will? Is free will compatible with a materialist approach to the study of behaviour? Is this truly free behaviour uniquely human?

I believe many of these problems arise from mixing up different perspectives on action. There are two ways to look at voluntary actions. If we want to study animals or humans who cannot communicate, then we have to define voluntary actions on the basis of behaviour (the third-person perspective). But, through introspection, we can also define voluntary actions in terms of experience (the first-person perspective). As we shall see, these two perspectives tell very different stories about voluntary actions.

## Volition from the third-person perspective

For an outside observer, the key feature of a voluntary act as opposed to a reflex is that the voluntary act cannot be fully predicted from the preceding context. The implication is that, if the behaviour is not being determined by external events, then the choices must be made ‘from inside’, endogenously.

### Unpredictable behaviour

The matter of predictability has played a major role in the design of many experiments in which attempts have been made to manipulate the degree of voluntariness associated with an action. For example, in one condition, the subject’s responses might be entirely determined by an external stimulus (*press the left key when the red light goes on*). In the other condition, subjects might be told, *press whichever key you like when the light goes on* (e.g. Frith et al. 1991). In this latter condition, each choice is not determined by any external stimulus and cannot easily be predicted. The actions in both conditions are clearly voluntary in the sense that subjects could choose not to follow the experimenter’s instructions, but, assuming that the instructions are obeyed, the second, unconstrained condition is clearly more voluntary and more ‘free’ than the first. Libet’s classic experiment (Libet et al. 1983) on ‘a freely voluntary act’ is based on the same principle, except here the precise *time* of the act that is chosen by the subject and that is difficult to predict.

When subjects have to make free choices, activity is typically seen in dorsolateral prefrontal cortex, a region that seems to be involved in selecting actions when there are a number of competing possibilities (Jenkins et al. 2000; Frith 2000). By definition, the greatest possible unpredictability is achieved by making choices at random, and indeed, random behaviour is perceived as being more ‘free’ (Ebert and Wegner 2011). Thus, since free choice is related to unpredictability, it is not surprising to find that performing a task in which subjects are specifically instructed to try and generate random sequences elicits activity in the same brain regions as tasks involving ‘free choice’ (Jahanshahi et al. 2000).

There are many reasons why behaving randomly may not seem a very good way of characterising volitional, free behaviour. For one thing, random behaviour does not seem to be a very rational way of dealing with the world. In addition, true randomness is extremely difficult to achieve either as a deliberate human endeavour or with mathematical aids. Is it possible for a nervous system to generate truly random behaviour?

As is so often the case, these intuitive ideas about randomness are quite wrong. There are many competitive situations where it is an advantage if others cannot predict what you are going to do next. Penalty shots in football are an obvious example, and random behaviour is optimal when playing zero-sum games such as stone–paper–scissors (Osborne 2004, p. 141). The advantages of random behaviour are even more obvious for non-human animals, including invertebrates (Brembs 2011). It is a great advantage to a predator if the behaviour of prey is predictable. For example, when fish perceive a sudden pressure change on one side of their body, they bend in a C-shape away from the perceived stimulus to escape in the opposite direction (C-start response). Taking advantage of this highly predictable behaviour, the tentacled snake elicits the C-start response with one part of its body causing the fish to jump into its mouth, which has been positioned in the predicted path of the fish (Catania 2009). Predictability is not a good strategy for survival, and many species have evolved unpredictable (protean) behaviour as a means of escape (Humphries and Driver 1970). For example, when an air movement is detected, a cockroach escapes away from that side. However, the precise angle taken by the animal cannot be predicted (Domenici et al. 2008).

If voluntary behaviour is defined in terms of unpredictability and flexibility, then such behaviour is present in many species, including invertebrates, and it follows that the central nervous system of these creatures has solved the problem of generating unpredictable behaviour (Brembs 2011). In humans, in association with the development of the prefrontal cortex, the ability to select responses in a flexible manner is greatly enhanced and, with some deliberate mental effort, can be applied to novel situations. It is this kind of flexible behaviour that is engaged in tasks like that of Libet in which subjects must freely decide when or how to respond (Frith 2000; Roepstorff and Frith 2004). Responding ‘freely’ in the Libet task can be interpreted as selecting, at random, one from of a range of different time intervals.

### Endogenous, self-generated behaviour

Unpredictable behaviour is unpredictable precisely because it cannot be predicted on the basis of prior external events. In this sense, the origin of the behaviour lies in the behaving individual rather than in the environment. The behaviour is self-generated or endogenous.

In terms of traditional stimulus–response psychology, such endogenous behaviour is difficult to explain, since this is a response with no eliciting stimulus. In contrast, within an engineering framework, such behaviour is perfectly natural since responses, better termed actions, are seen as inputs into the environment, which elicit changes, that is, outputs from the environment. Through such actions, we learn about the environment.

Such behaviour is captured within the framework of *instrumental conditioning*. In instrumental, or operant, conditioning, the behaviour is determined by the expectation of a valuable outcome. The most famous example of operant condition is the Skinner box. Here, the behaviour of a pigeon pecking a disc is maintained by the *consequences* of this action, receiving food. Through instrumental conditioning, an organism learns that different actions are associated with different outcomes. The action chosen is that associated with the highest expected outcome in the current context (Sutton and Barto 1998). Such choices are self-generated in the sense that they are determined by internal representations of expected values. These representations have been formed through previous interactions with the environment, but the current choice is not determined by some specific environmental stimulus. Instrumental learning is sometimes described as dealing with the modification of ‘voluntary behaviour’. Behaviour that is self-generated in this sense is a feature of many animal species and is certainly not uniquely human.

When we choose an action of the basis of the expected outcome, our action may be determined endogenously, but it is still determined. We choose the action associated with the highest expected value for its outcome. In this sense, our action is rational, but it is also highly predictable and, in that sense, not ‘free’. There is, however, another parameter associated with models currently popular for explaining decision-making. This is the explore/exploit parameter (see e.g. Cohen et al. 2007). At one extreme of this parameter, animals simply *exploit* the knowledge they have acquired, always choosing the action associated with the highest expected value. This is *resource* acquisition. At the other extreme, animals do not choose the action with the highest value, but *explore* what happens if other options are chosen. This is *information* acquisition. The explore/exploit parameter is particularly relevant when animals are foraging for sources of food. Is it better to go to a known food source (exploit), or is it better to explore in the hope of finding a new and possibly better source? Some exploration will be beneficial since a) the food supply in the known location may eventually be exhausted and b) without exploration, we may remain trapped in a local minimum (to put it in mathematical terms) and never discover the better location elsewhere.

For social animals, such as bees, it is an advantage for the group to have a proportion of individuals engaged in exploration. In this way, the group can fully exploit known food sources, but also identify new and better sources. In bees, there are genetically determined individual differences in the propensity to explore, linked to the dopamine system. This observation suggests an interesting analogy with novelty-seeking behaviour in humans (Liang et al. 2012). However, the propensity to explore is also affected by the environment. When resource levels are high, exploration is more likely to be chosen because, in such an environment, this option is less risky (Eliassen et al. 2007).

Exploratory choices are less determined than exploitative choices. Indeed, the algorithms used to model such choices typically include a random element for making the exploratory selection. However, the ability to make exploratory choices is clearly not uniquely human and is therefore not relevant to an understanding of free will. On the other hand, this framework suggests that decision-making and hence volition should be conceived of as a hierarchy. At a low level, choice is made between different actions. At a higher level, the decision is taken whether such choices should be made in an exploitative or an exploratory mode. For humans, at least, there is indirect evidence that the choice to explore depends upon overriding exploitative tendencies and is linked to activity in frontopolar cortex (Daw et al. 2006). Exploratory behaviour in humans would then be another example of the highly flexible volitional behaviour associated with the evolution of the prefrontal cortex.

### Predicting endogenous behaviour

Ever since Libet’s innovative experiment, there has been great interest in discovering the extent to which it is possible to predict someone’s behaviour from the brain activity preceding the action. For example, Soon et al. (2008) used fMRI to show that brain activity occurring several seconds prior to the movement predicted better than chance^1^ the choice that subjects would make when the decision to press with the left or the right finger was freely chosen. I have argued above that tasks of this sort are problematic since, in essence, the experimenter is asking her subjects to try to be unpredictable and random (Roepstorff and Frith 2004). To achieve such behaviour depends on a complex and underspecified cognitive process.

More straightforward paradigms can be used to contrast actions that are controlled endogenously and exogenously. In typical discrimination tasks, participants choose which response to make on the basis of sensory information. For example, Bode et al. (2012) showed participants pictures of pianos or chairs. The participants indicated which object they thought had been presented by pressing keys with the left or right finger. However, the discrimination was made difficult by masking the pictures, and on some trials, no picture was actually present between the masks. When enough sensory information is present to discriminate the pictures, the choice is determined exogenously by brain events that occur immediately after the presentation of the stimulus. When, however, there is no sensory information available, the choice must be made endogenously, even though the participant is not aware of this. These expectations were confirmed by measuring EEG during the performance of the task and using this activity to predict the choice made. When sensory information was available, the choice could only be predicted from brain activity occurring after stimulus presentation. In contrast, when no sensory information was available, the choice could be predicted from brain activity occurring prior to stimulus presentation and therefore also prior to the making of the decision. This activity derived from the participant’s previous choice history.

The authors suggest that, at the beginning of each trial in this object classification task, possible choices are primed on the basis of the previous choice history. If sensory information about the object becomes available, then this priming is overridden and choice is determined by this new information. In free choice tasks, where there is no exogenous information, choice is determined, to some degree, by what has happened in the past and hence prior brain activity can predict choice better than chance.

There is direct evidence that unconscious primes can influence behaviour in a free choice task. For example, Kiesel et al. (2006) used a two-choice reaction time task in which some choices were fixed, since a visual stimulus (4 or 6) indicated which choice should be made, while other trials were free since the stimulus (0) indicated that participants should choose for themselves which choice to make. The stimuli were preceded by subliminal stimuli (also 4 or 6), which primed subjects to choose one or other option. There was a significant, though small effect (~5 %) of these subliminal primes on the choice made in the free trials. Thus, although there are effects of unconscious primes on free choices, these biases are often overcome. These priming effects are too small to prove that our actions, whether called voluntary or involuntary, are completely determined by our previous history.

When we look at human behaviour from a third-person perspective, we can see that it is continuous with behaviour in animals. Many creatures perform acts that are volitional in the sense that these acts are not determined by immediate signals in the environment and are therefore difficult to predict. Humans are certainly more flexible in their ability to generate unconstrained acts, but the basic mechanisms for doing this were already in place. If we are to identify unique aspect of human volition, then we need to turn to the first-person perspective. Indeed, I believe that it is only by taking into account the first-person perspective that we can properly understand volition.

## Volition from a first-person perspective

As well as observing what people do from a third-person perspective, we can also ask them about their experience of action. This is the first-person perspective on volition. As soon as we take the first-person perspective on volition, we restrict ourselves to the study of humans. To collect evidence from a first-person perspective, we need participants who are, first, able to reflect upon their actions and, second, are able to communicate the result of such reflection to others. These abilities may well be uniquely human (Frith 2012).

### The experience of action

Metzinger (2006) has described the experience of volition as ‘thin and evasive’. This is because we are rarely aware of the sensory consequences of our own actions. For example, participants can be given misleading visual feedback about the position of their limb when they are controlling the position of a cursor on a screen. In order to move the cursor straightforward, they may have to move their hand slightly to one side. People are unaware of such deviations as long as they are less than about 15 degrees (Fourneret and Jeannerod 1998). At the sub-personal level (Hornsby 2000), the brain takes account of the deviation since the movement is appropriately adjusted to ensure that the cursor reaches the target. It seems plausible that the sensory consequences of action do not normally enter awareness because they are predicted on the basis of the commands sent to the motor system (Frith et al. 2000). If everything happens as predicted, the resultant sensations are uninformative and need not be at the focus of attention. This is why self-tickling generates such minimal and unexciting sensations. On the other hand, even a small distortion of the sensory stimulation generated by self-tickling reinstates the tickling sensation (Blakemore et al. 1999).

We may have little awareness of the sensations associated with our actions, but, on the other hand, we are vividly aware of acting on the world. *So what is it about our actions that we are aware of?* I suggest that, when all is going well, we are simply aware of our intention to act and of the effect of our action on the world (the outcome) as a check that our intention has been fulfilled. This idea is implicit in Libet’s famous experiment on voluntary action (Libet et al. 1983). Participants were asked to lift their finger ‘whenever they had the urge to do so’. They were also asked to introspect either on when they had the urge to act (the intention) or on when the finger movement occurred (the outcome of the intention).

The idea that we are only aware of the intention to act and its outcome can make sense of otherwise extremely strange abnormalities of awareness of volition that can sometimes occur as a consequence of brain damage. After damage to the right hemisphere, including the parietal cortex, some patients develop *anosognosia for hemiplegia* (Bisiach et al. 1986); that is, they are unaware that their left limb is paralysed and claim that they can and do move it. This experience can be explained as follows. The patients’ motor systems are intact in as far as they can formulate an intention to move the paralysed limb. In the normal case, this intention would be followed by the outcome of the limb moving. The failure of the limb to move would normally indicate that something was wrong with the system. In the case of anosognosia, however, the feedback, indicating that the limb has not moved, fails to register. As a result, patients experience the intention to move and, in the absence of any evidence to the contrary, assume that the movement has taken place. Fotopoulou et al. (2008) have recently obtained empirical evidence in favour of this account. This explanation requires that the visual feedback, indicating that the limb has not moved, is not registered. This lack of registration is probably caused by right parietal lobe damage and the neglect of visual signals from the left side of space often associated with such damage (Driver and Vuilleumier 2001).

### The experience of agency

In a series of very ingenious experiments, Haggard et al. have demonstrated that there is an intimate connection between these two key aspects of our experience of action: the intention and its outcome. It is this connection that is the basis of our experience of agency, that is, of being the cause of the outcomes of our actions. Haggard et al. studied simple actions with consequences, for example pressing a button followed after 250 ms by a tone. Using the technique pioneered by Libet et al. (1983), participants are asked to indicate the time at which they press the button and, on other trials, the time at which they hear the tone. The first study in this series (Haggard et al. 2002) uncovered the phenomenon of *intentional binding*. Participants experience their action and its outcome as being closer together in mental time than they are in physical time. Thus, the button press is experienced as being slightly later in mental time than in physical time, while the tone is experienced as occurring slightly earlier. This binding effect is reversed if the movement preceding the tone is involuntary, being caused by transcranial magnetic stimulation (TMS). The involuntary movement and its ‘outcome’ are experienced as being further apart in mental than in physical time.

I suggest that the binding phenomenon at the heart of our experience of agency is essentially the same as the binding that occurs when we bring together colour, shape and movement in the perception of an object (Treisman 1996). In both cases, the effect of the binding, which happens at a sub-personal level (Di Lollo 2012), is to create a single phenomenological entity. The entity that forms our experience of agency is an action containing both an intention and an outcome. In consequence, our experience of this action is affected, not only by factors that exist before the action starts, but also by what happens after the action has been initiated (Moore et al. 2009). First, there is a predictive component. The time at which an action is experienced as being initiated is affected by prior expectations about the likelihood of the outcome. The time of initiating the action is perceived as later, if the outcome has been experienced as occurring 75 %, rather than 50 % of the time. Second, there is a post-dictive effect. In the case of a 50 % probability of the outcome, the time of initiating the action is experienced as occurring later in the cases in which it is followed by an outcome. Note that, in this example, the time at which people experience initiating an action is affected by an event that happens hundreds of milliseconds after the initiation.

I suggest that, as with all perception, the perception of agency depends on an (unconscious/sub-personal) inference based on prior expectations and on evidence from the senses (Helmholtz 1878). Expectations about agency can be learned through the repeated experience of contingencies between intentions and outcomes, as in the experiment by Moore et al. (2009) just discussed, but they can also be derived from instructions. For example, participants in an experiment conducted by Dogge et al. (2012) were encouraged to experience themselves as the cause of a tone, even though the movements they made were involuntary. These instructions significantly increased the strength of intentional binding.

As yet, little is known concerning the sensory evidence used to construct the sense of agency. However, one of the key sources of evidence seems to be the fluency with which the action is selected, rather than the quality of the subsequent performance. An action can be more easily and fluently selected after it has been primed. Participants experienced greater control over action effects when the action had been compatibly versus incompatibly primed (Chambon and Haggard 2012), even though they were unaware of these subliminal primes. I have already mentioned that subliminal action primes can influence behaviour in a free choice task (Kiesel et al. 2006). Subliminal action primes also increase the sense of agency associated with compatible choices in a free selection task (Linser and Goschke 2007), even though, in a sense, less ‘will’ is being exerted when participants choose the primed action.

Further evidence for the role of expectation and inference in the experience of agency is provided by the various illusions that have been demonstrated. Such illusions can occur if strong expectations about contingency are violated. For example, after repeated exposure to a scenario in which a light flash usually occurred 135 ms after a key press, participants experienced a light flash that occurred 35 ms after their key press as occurring before the key press (Stetson et al. 2006).

Wegner et al. have shown that an illusory experience of agency can arise merely because of a contingency between the intention to act and an appropriate outcome. Using a modern version of the Ouija Board, participants believed that they had moved a cursor towards a location that they had just been primed to think about, even though the movement had actually been caused by a stooge (Wegner and Wheatley 1999). The opposite illusion can also occur when people falsely believe they are not the agent of an action. This effect sometimes emerges during the use of *facilitated communication* (Wegner et al. 2003). This technique was developed to aid communication in the handicapped. A facilitator detects small movements in the hand of the person who is answering questions. For example, the hand might rest on a keyboard, but be too weak to press the keys. The facilitator amplifies these movements to generate the key presses desired by the communicator. The problem with this technique is that facilitators will firmly believe that they are reporting the answers of the communicator, even when the ‘communicator’ is a stooge who is making random movements (Wheeler et al. 1993).

That the experience of agency and volition is inferred from various sensory cues and prior expectations may account for the different ways in which the term agency is used. The various versions of the term place emphasis on different aspects of the experience. Libet (1985) emphasises precursors of action (the decision to act or not), Wegner (2005) emphasises the feeling of authorship (the sensation of being in control), Metcalfe and Son (2012) emphasise the judgement of being in control (the inference of being in control), while many (e.g. Aarts et al. 2005) emphasise the relation between action and outcome (having an effect on the world). As I shall argue below, the way we discuss the experience of agency is not simply a matter of terminology and emphasis. It can also have an effect on the way agency is experienced.

## Agency, free will and responsibility

Whatever the precise mechanisms underlying its perception, volition, seen from the first-person perspective, is associated with a vivid experience of agency. As agents, we have the experience of desiring an outcome and of choosing the action that will achieve it. Some believe that it is this experience of being an agent that leads to a belief in free will. For example, Spinoza (1677) said, ‘Experience teaches us no less clearly than reason, that men believe themselves to be free, simply because they are conscious of their actions, and unconscious of the causes whereby those actions are determined’. The implication is that this belief about free will is an illusion, a supposition supported by many, from Hume (1758) to Wegner (2003).

In contrast to this approach, I suggest that the important feature of our awareness of agency is that it gives us a feeling of responsibility. I believe that research on volition will prove more tractable if we look at it in terms of responsibility rather than free will. My approach relates to earlier philosophical ideas, particularly those associated with Epicurus.

### Responsibility, agency and regret

According to Epicurus, we acquire the idea that we are causal agents through the observation that human beings, including ourselves, are praised and blamed for their actions. Epicurus believed that our feeling of responsibility for our actions has two sources. First, there is the feeling that it was ‘us ourselves’^2^ rather something else that caused the action. Second, there is the feeling that we could have chosen another action (Bobzien 2006).

The feeling that it was us ourselves that caused the action seems very close to the experience of agency discussed in the last section. The relationship between responsibility and agency has long been recognised and, more recently, has been studied experimentally. When asked about free will and responsibility, most people will respond, first, that we are only responsible for behaviour that is caused consciously (i.e. associated with a sense of agency), rather than unconsciously (Shepherd 2012) and, second, that the more an actor is identified with an action, the more appropriate it is that responsibility should be assigned to that actor (Woolfolk et al. 2006). When the experience of agency is measured directly using Haggard’s intentional binding paradigm, there is a greater sense of agency (i.e. more binding) when the consequences have a moral rather than a merely economic content (Moretto et al. 2011).

The second basis for responsibility, proposed by Epicurus, is the feeling that we could have done otherwise. It is this feeling that creates regret. ‘For it is on the grounds that it was possible for us also not to have chosen and not to have done this that we feel regret’.^3^ Regret must be distinguished from disappointment. We experience disappointment when the outcome of our action is worse than we expected. In contrast, we experience regret when we discover that an action that we could have chosen, but didn’t, would have obtained a better outcome than the one we actually chose.

The intensity of the regret we feel does not simply depend on the difference between what we achieved and what we might have achieved. It also depends on the extent to which we perceive ourselves to be the agent of the choice. For example, people believe that they would feel more regret if the regretted action was atypical, rather than habitual, or if the regretted action was one of commission, rather than omission (Guttentag and Ferrell 2004). Also, people report feeling more regret if the regretted action was the result of an individual decision rather than the majority decision of eight people, even though their choice conformed with the majority (Nicolle et al. 2011).

Feeling regret depends upon counterfactual reasoning. We are reflecting on what would have happened, if we had acted differently. We can also anticipate regret by reflecting, in advance of the decision, on how we would feel if it turns out that we have made the wrong choice. Anticipated regret affects choice behaviour. This has been shown with auctions. In an auction, I will feel disappointment if my bid does not acquire the item I want. On the other hand, if I learn that my bid was only just below the winning bid, I will feel regret for not having bid higher. As a result, people make higher bids when they know that they will be told the winning bid if they lose, in comparison with auctions in which they are given no feedback about the other bids (Filiz-Ozbay and Ozbay 2007). Taking account of anticipated regret seems to require intact orbitofrontal cortex (Camille et al. 2004).

### Responsibility and punishment

The concept of responsibility for action is of great importance, since, in most legal systems, only people who are responsible for their actions deserve punishment (see e.g. Goodenough 2004). In its turn, punishment is important because it has a critical role in stabilising society. For example, experiments in behavioural economics demonstrate the importance of punishment in maintaining cooperation in social groups. Fehr and Gachter (2002) showed that cooperation flourishes if altruistic punishment is applied to defectors by other members of the group, but breaks down if punishment is not possible. Because of the advantages of cooperation, in the long run people will choose to be part of an institution in which punishment is sanctioned, rather than one in which it is not (Gürerk et al. 2006).

However, the key point to be made here is that, at least in the setting of an economic game, punishment is only applied to those players who are considered to be responsible for their actions. Punishment is not applied if participants in the game have been told that a player is simply following printed instructions, even though the participant loses just as much money when confronted with the intentional and the non-intentional player (Singer et al. 2004). Given the importance of distinguishing between intentional and unintentional acts, it is not surprising that the ability to make such a distinction seems to emerge very early, at between 6 and 9 months of age (Behne et al. 2005). Infants of 9 months reacted with more impatience when they failed to get a toy because it was withheld deliberately, rather than accidentally dropped.

## The emergence of responsibility

I believe that, by discussing volitional behaviour in terms of responsibility rather than free will, empirical explorations about the emergence of such behaviour become much more tractable. For example, at various times in history, animals from insects to pigs were tried in court for their misdemeanours (Humphrey 2002), but today we take it for granted that non-human animals are not responsible for their actions. Likewise, we take it for granted that very young children are not responsible for their actions. The implication of these observations is that responsibility must emerge at some point both in evolution and in individual development.

When, precisely, this point is reached in development remains controversial. There are wide differences in the minimum age of criminal responsibility between different legal systems varying from 7 years (e.g. Switzerland) to 18 years (e.g. Luxembourg, see Hazel 2008). There is currently much debate concerning the possibility that adolescents are not fully responsible for their actions since, at this age, the frontal lobes are still not fully mature (see e.g. Mackintosh 2011).

This variability may relate to observation that different aspects of responsibility emerge at different ages. Only by the age of 5 can children report a mature experience of agency, distinguishing between a voluntary movement of the leg and a knee jerk reflex (Shultz et al. 1980). A true experience of regret seems to arise much later, at around the age of 9 (Rafetseder and Perner 2012). Prior to this age, children base their judgements solely on what they got without taking into account what they could have got. Finally, there is some preliminary evidence that the excessive risk-taking behaviour seen in adolescence may be related to a lack of, or failure to take account of, anticipated regret (Gerrard et al. 1996). Thus, it seems that the ability to take regret into consideration, when making choices, continues to develop during adolescence (Habib et al. 2012).

### The creation of responsibility

An obvious problem, when we take the first-person perspective in our studies of volition, is that we need our participants to be able to communicate to us their feelings and experiences. Is this the reason why we do not assign responsibility to non-human animals and non-verbal infants? Is it that animals and infants have a rich experience of volition, but simply cannot tell us about it? This seems to me unlikely. The reports and behaviour of young children suggest that their experience of volition is different from that of adults. I suggest, therefore, that the ability to report and discuss the experience of action is not simply a window on volition. Rather, these explicit meta-cognitive abilities help to create our experience of agency and responsibility. In part, this is because such discussions typically involve reflecting on what might have happened if we had acted otherwise. Counterfactual reasoning is thereby used to create a sense of responsibility. The experience of agency and responsibility is found only in humans, and only above about 5 years of age, because the emergence of such experience depends on the uniquely human faculties of counterfactual reasoning and explicit meta-cognition (Frith 2012).

Epicurus anticipated these ideas through his suggestion that we acquire the idea that we are causal agents through the observation that human beings, including ourselves, are praised and blamed for their actions (Bobzien 2006). In other words, how we experience volition is something that can be learned. As yet, there have been few empirical studies of this proposal, but I believe that what little evidence there is supports the idea. For example, I have already mentioned the study by Dogge et al. (2012) showing that intentional binding between involuntary actions and outcomes can be increased by teaching the participants to consider themselves as the cause of the effect, even when they are not.

A recent study reveals that instructions about the nature of action can also change behaviour. Job et al. (2010) told one group of participants, ‘Working on a strenuous mental task can make you feel tired such that you need a break before accomplishing a new task’ (limited resource condition), while another group were told, ‘Sometimes, working on a strenuous mental task can make you feel energized for further challenging activities’ (non-limited resource condition). These instructions altered behaviour such that the limited resource group made more errors on a STROOP task after performing a strenuous mental task, while the non-limited resource group made fewer errors.^4^

My interpretation of this result is based on the idea that introspection is a very unreliable guide to the cognitive processes that underlie our behaviour (Nisbett and Wilson 1977; Schwitzgebel 2008). It is becoming increasingly apparent that we are wrong if we think that, by consulting our intuitions, we can uncover the basic components of the cognitive engine that drives our behaviour and distinguish between the ‘basic model’ and the ‘optional extras’ that are formed by experience (Danziger 2006). After performing a strenuous task, our experience of action will change, but we will not be sure about what these changed sensations mean. As a result of this uncertainty, discussion with others can alter our interpretation and our response to our experiences of action. The experiment by Job et al. (2010) suggests how instructions might create an ‘optional extra’ for our experience of mental effort.

It follows from this argument that our experience of action should be subject to the effects of culture. There is some evidence that this is the case for the feeling of responsibility. Among the Mopan Mayas of Central America, for example, both children and adults are punished according to the outcome of their actions. Punishment is applied equally if the outcome is accidental, rather than deliberate. As a result, the defence of ‘I didn’t mean it!’ is considered irrelevant and is seldom attempted. This disregard for the mental states of actors is found at an institutional level and in everyday gossip. Saying a falsehood is not excused even if, at the time, the speaker believes it to be true, and children who indulge in pretend play are reproved (Danziger 2006). The experience of responsibility in this culture appears to be different from the experience in our culture.

## Is there anything unique about human volition?

Volition looks very different from the first-person compared with the third-person perspective. From the third-person perspective, human volitional behaviour is clearly on a continuum with volitional behaviour in other species. Many creatures, including invertebrates, behave in ways that are not totally constrained by the immediate environment. In other words, the behaviour is generated ‘from inside’. Many creatures can behave unpredictably and even randomly. Humans differ only in that they have a greater flexibility in the endogenous generation of behaviour, especially in relation to novel behaviour. From this point of view, the idea of a uniquely human form of volition involving free will does not seem very plausible.

In contrast, at least in contemporary western cultures, when we take a first-person perspective, we are often vividly aware of being the author of and therefore responsible for our actions. We can also be acutely aware of making choices and strongly believe that we could have done otherwise. This difference between first- and third-person perspectives also influences our attitude to our own actions and those of others. Each of us believes that our own lives are more driven by free will than are the lives of others (Pronin and Kugler 2010): my behaviour is driven by my intentions and desires, while yours is driven by your personality, the situation and various unconscious biases. It is the first-person aspect of volition that is uniquely human. We can only study this aspect of volition because people can describe their experiences. This is an ability found only in humans.

As I already mentioned, I do not believe that non-human animals have a first-person experience of agency, but are not able to tell us about it. I believe that they have no experience of agency. This is because the experience of agency is created by discussion with others. We do not have direct sensations of agency derived from specialised receptors. Agency is something we experience on the basis of prior expectations and low-level sensations, such as selection fluency and kinaesthetic feedback. There is evidence that such signals are also available to non-human animals, since many animals show preferences for actions that have an effect. For example, mice prefer to press a lever that moves rather than one that is stationary (Kish and Barnes 1961), and monkeys prefer to press a lever that turns on a light rather than one that has no effect (Moon and Lodahl 1956). However, the availability of such signal is not sufficient to create an experience of agency. The experience of agency requires that these signals be interpreted. Research is needed to identify more precisely the various kinds of sensory evidence that provide the basis for the experience of agency. It will also be important to know more about how malleable our interpretations of these signals can be. For example, no amount of discussion can change our experience that the lines in the Muller-Lyer illusion are of the same length (Pylyshyn 1999), but our experience of agency seems not to be so impenetrable. For example, there is the study from Dogge et al. (2012), mentioned above, in which the experience of intentional binding could be altered by suggestions that an involuntary action was causing an effect.

Can we be taught to reinterpret the sensory evidence associated with action? Consider, for example, the phenomenon through which we like something we have seen before even though we cannot remember seeing it before (Kunst-Wilson and Zajonc 1980). The assumption, in this case, is that the sensation of perceptual fluency associated with something we have just seen is taken as evidence of liking, rather than familiarity (Bornstein and D’Agostino 1994). Could we learn to experience this fluency as familiarity? We need to explore further the mechanisms by which discussions and instructions can alter our experience of perception and action (as in Job et al. 2010).

### Culture and the experience of action

If our experience of action can be altered by teaching and instructions, then we would expect that the experience of agency might be different in different cultures. Indeed, as we have seen, there is some preliminary evidence for this (Danziger 2006), based on the way in which agency and responsibility are talked about and on how punishment is linked to action in the Mopan Mayas. It would be very interesting to see whether experimental measures, such as intentional binding, also indicated differences in the experience of agency in this culture.

I have previously suggested that the effects of culture on the experience of action depend on two mental phenomena (Frith 2012): first, that we can reflect on our experiences and positively enjoy discussing them with others and second, that our introspections about these experiences are very unreliable (Nisbett and Wilson 1977; Schwitzgebel 2008). It is precisely because of this unreliability of introspection that experiences can be changed through discussion.

One effect of discussion could be to make our introspection more accurate and reliable. For example, Bahrami et al. (2010) showed, through discussion of their visual experience, two people could arrive at a more accurate representation of the world than the best working on his own. In the same way, through discussion with others, we might come to a more accurate account of the sources of our actions than we can achieve on our own. This idea seems plausible, given that others may be more aware, than we are ourselves, of the various biases that affect our own behaviour (Pronin and Kugler 2010).

However, the effects of culture on our experience need not necessarily lead to a more accurate account of the cognitive processes that generate our actions. For example, we can doubt whether our experience of being a causal agent and, hence, of being responsible for the outcomes of our actions accurately reflects the processes underlying our actions. Nevertheless, this experience is extremely important for generating social cohesion and advantages for the group. Perhaps, in this case, we should dispense with accuracy.

## Footnotes

1 However, the prediction accuracy was only just better than chance at about 60 %.

2 (‘the cause from ourselves’, ‘that through ourselves’, Bobzien 2006).

3 (Alexander of Aphrodisias, *On Fate*, Bobzien 1998).

4 Telling students that free will is an illusion also changes their behaviour. They are more likely to cheat on a test (Vohs and Schooler 2008).
